# Supplementary material for: Cryopreserved Tissue Biospecimens Offer Superior Quality for Whole-Genome Sequencing of Various Cancers Compared to Paired Formalin-Fixed Paraffin-Embedded Tissues
Source: Int J Mol Sci. 2025 Nov 14;26(22):11038. doi: 10.3390/ijms262211038 (PMC12652624; doi:10.3390/ijms262211038)
Supplement: Supplementary file 1 [file ijms-26-11038-s001.zip › Supplemental Tables S2 and S3.pptx]

## Slide 1
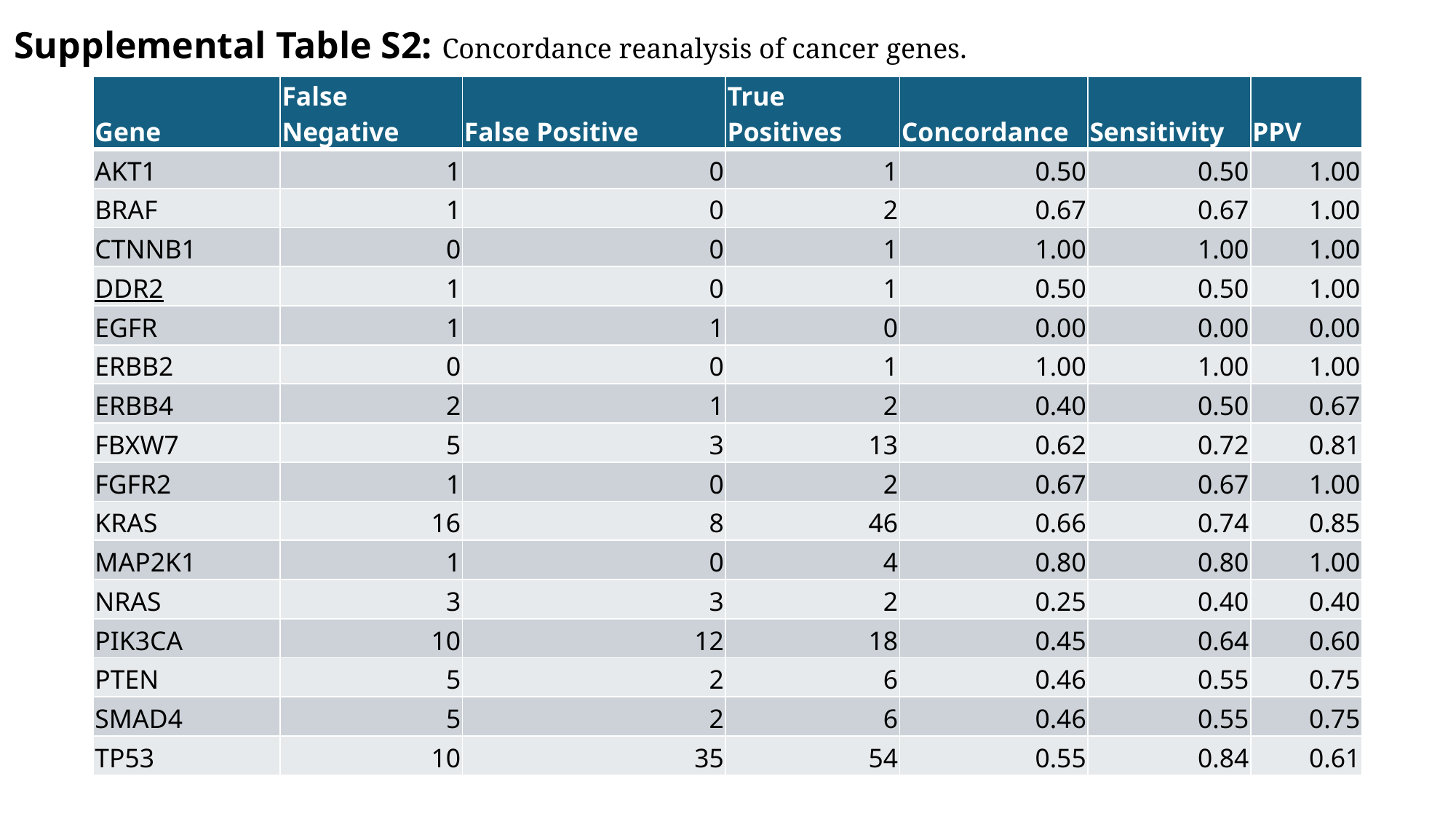

Supplemental Table S2: Concordance reanalysis of cancer genes.
| Gene | False Negative | False Positive | True Positives | Concordance | Sensitivity | PPV |
| --- | --- | --- | --- | --- | --- | --- |
| AKT1 | 1 | 0 | 1 | 0.50 | 0.50 | 1.00 |
| BRAF | 1 | 0 | 2 | 0.67 | 0.67 | 1.00 |
| CTNNB1 | 0 | 0 | 1 | 1.00 | 1.00 | 1.00 |
| DDR2 | 1 | 0 | 1 | 0.50 | 0.50 | 1.00 |
| EGFR | 1 | 1 | 0 | 0.00 | 0.00 | 0.00 |
| ERBB2 | 0 | 0 | 1 | 1.00 | 1.00 | 1.00 |
| ERBB4 | 2 | 1 | 2 | 0.40 | 0.50 | 0.67 |
| FBXW7 | 5 | 3 | 13 | 0.62 | 0.72 | 0.81 |
| FGFR2 | 1 | 0 | 2 | 0.67 | 0.67 | 1.00 |
| KRAS | 16 | 8 | 46 | 0.66 | 0.74 | 0.85 |
| MAP2K1 | 1 | 0 | 4 | 0.80 | 0.80 | 1.00 |
| NRAS | 3 | 3 | 2 | 0.25 | 0.40 | 0.40 |
| PIK3CA | 10 | 12 | 18 | 0.45 | 0.64 | 0.60 |
| PTEN | 5 | 2 | 6 | 0.46 | 0.55 | 0.75 |
| SMAD4 | 5 | 2 | 6 | 0.46 | 0.55 | 0.75 |
| TP53 | 10 | 35 | 54 | 0.55 | 0.84 | 0.61 |

## Slide 2
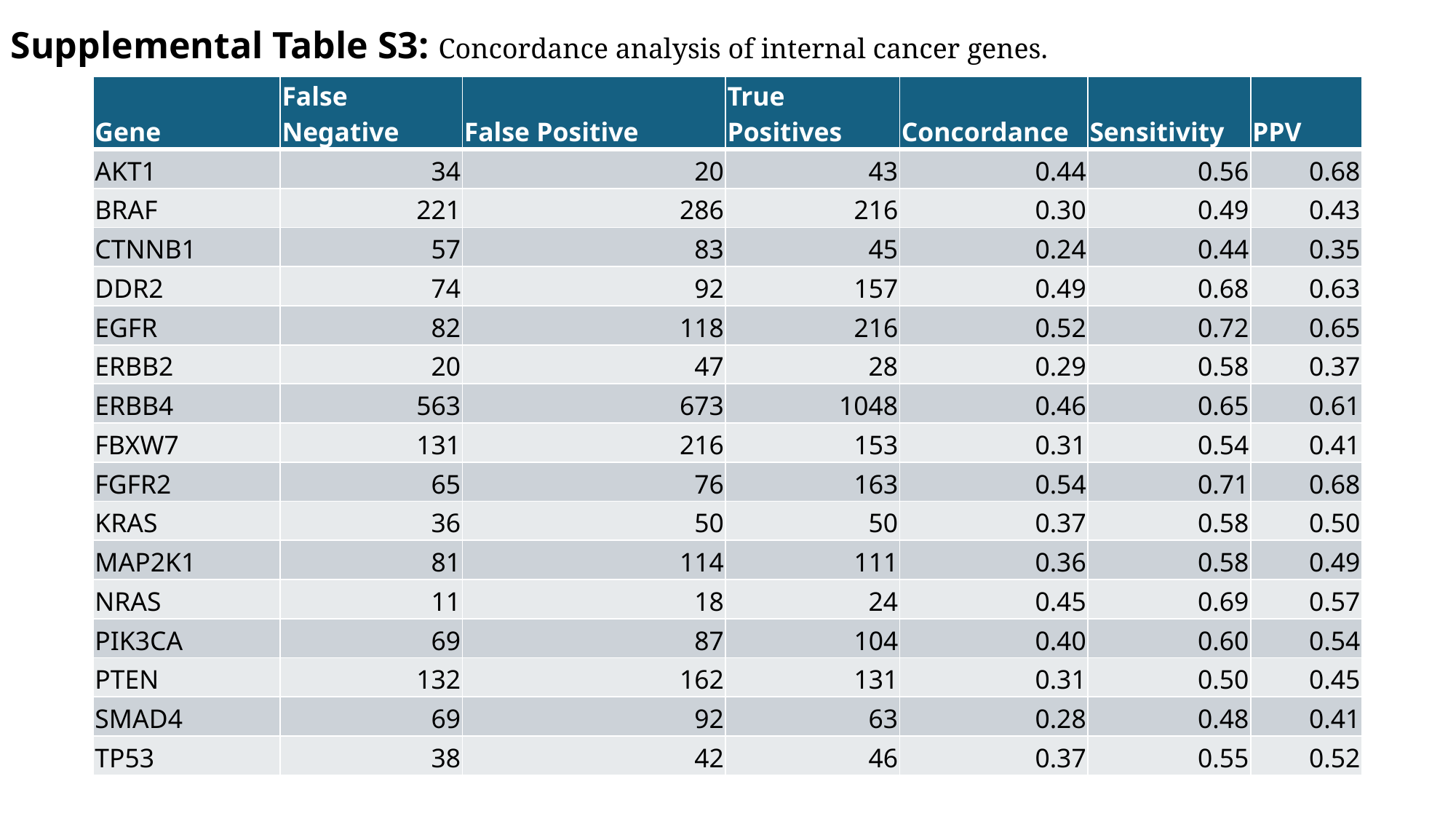

Supplemental Table S3: Concordance analysis of internal cancer genes.
| Gene | False Negative | False Positive | True Positives | Concordance | Sensitivity | PPV |
| --- | --- | --- | --- | --- | --- | --- |
| AKT1 | 34 | 20 | 43 | 0.44 | 0.56 | 0.68 |
| BRAF | 221 | 286 | 216 | 0.30 | 0.49 | 0.43 |
| CTNNB1 | 57 | 83 | 45 | 0.24 | 0.44 | 0.35 |
| DDR2 | 74 | 92 | 157 | 0.49 | 0.68 | 0.63 |
| EGFR | 82 | 118 | 216 | 0.52 | 0.72 | 0.65 |
| ERBB2 | 20 | 47 | 28 | 0.29 | 0.58 | 0.37 |
| ERBB4 | 563 | 673 | 1048 | 0.46 | 0.65 | 0.61 |
| FBXW7 | 131 | 216 | 153 | 0.31 | 0.54 | 0.41 |
| FGFR2 | 65 | 76 | 163 | 0.54 | 0.71 | 0.68 |
| KRAS | 36 | 50 | 50 | 0.37 | 0.58 | 0.50 |
| MAP2K1 | 81 | 114 | 111 | 0.36 | 0.58 | 0.49 |
| NRAS | 11 | 18 | 24 | 0.45 | 0.69 | 0.57 |
| PIK3CA | 69 | 87 | 104 | 0.40 | 0.60 | 0.54 |
| PTEN | 132 | 162 | 131 | 0.31 | 0.50 | 0.45 |
| SMAD4 | 69 | 92 | 63 | 0.28 | 0.48 | 0.41 |
| TP53 | 38 | 42 | 46 | 0.37 | 0.55 | 0.52 |
